# The Safety and Efficacy of Human Umbilical Cord-Derived Mesenchymal Stem Cells in Patients With Heart Failure and Myocardial Infarction: A Meta-Analysis of Clinical Trials

**DOI:** 10.7759/cureus.49645

**Published:** 2023-11-29

**Authors:** Mohamed R Abouzid, Karim Ali, Ibrahim Kamel, Sadaf Esteghamati, Amr Saleh, Mohammed Ghanim

**Affiliations:** 1 Internal Medicine, Baptist Hospitals of Southeast Texas, Beaumont, USA; 2 Internal Medicine, Hennepin Healthcare, Minneapolis, USA; 3 Internal Medicine, Steward Carney Hospital, Boston, USA; 4 Internal Medicine, University of La Verne, La Verne, USA; 5 Faculty of Medicine, Mansoura University, Mansoura, EGY; 6 Internal Medicine, University Hospital Sharjah, Sharjah, ARE

**Keywords:** mortality, ejection fraction, meta-analysis, regenerative therapy, stem cell therapy, myocardial infarction, heart failure, human umbilical cord-derived mesenchymal stromal cells

## Abstract

Evidence from preclinical and clinical studies suggests that human umbilical cord-derived mesenchymal stromal cells (HUC-MSCs) may be useful in treating heart failure and acute myocardial infarction (MI). However, the effects of stem cell therapy on patients with heart failure remain the subject of ongoing controversy, and the safety and effectiveness of HUC-MSCs therapy have not yet been proven. To date, there has been no systematic overview and meta-analysis of clinical studies using HUC-MSCs therapy for heart failure and MI. The purpose of this study is to assess the safety and efficacy of HUC-MSC therapy versus a placebo in patients with heart failure and MI.

While preparing this systematic review and meta-analysis, we adhered to the Preferred Reporting Items for Systematic Reviews and Meta-Analyses guidelines. A computer literature search of PubMed was performed. We considered randomized controlled trials (RCTs) that reported data on the safety and efficacy of HUC-MSC transplantation in patients with heart failure and MI. Two investigators independently searched the literature, extracted data, and rated the quality of the included research. Pooled data were analyzed using the fixed-effect model or the random-effect model in Review Manager 5.3. The Cochrane risk of bias tool was used to assess the bias of included studies. The primary outcome was ejection fraction (EF), whereas the secondary outcomes were readmission and mortality rates.

Three RCTs (201 patients) were included in this meta-analysis. The overall effect did not favor either of the two groups in terms of risk of readmission (risk ratio = 0.5, 95% confidence interval (CI) = 0.22-1.15, p = 0.10) as well as mortality rate (risk ratio = 0.44, 95% CI = 0.14-1.44, p = 0.18). However, there was an improvement in EF in patients who received HUC-MSCs compared to placebo after 12 months of transplantation (mean difference (MD) = 3.21, 95% CI = 2.91-3.51, p < 0.00001). At the six-month follow-up period, there was no significant improvement in EF (MD = 1.30, 95% CI = -1.94-4.54), p = 0.43), indicating that the duration of follow-up can shape the response to therapy.

Our findings indicate that HUC-MSC transplantation can improve EF but has no meaningful effect on readmission or mortality rates. Existing evidence is insufficient to confirm the efficacy of HUC-MSCs for broader therapeutic applications. Therefore, additional double-blind RCTs with larger sample sizes are required.

## Introduction and background

Cardiovascular diseases (CVDs) are a global health burden of grave proportions [1,2]. CVDs are a collection of diseases and injuries that affect the cardiovascular system and its supporting tissues. The most prevalent CVDs encompass (but are not limited to) coronary artery disease, congestive heart failure, ischemic and non-ischemic cardiomyopathy, and peripheral arterial disease. In the past 50 years, CVD treatment has made tremendous and unparalleled strides, as evidenced by a substantial and ongoing decline in CVD-related mortality [3]. Despite this progress, CVDs remain the primary cause of disability and mortality including premature death in the United States and all over the world, impacting 85.6 million Americans and accounting for 1 in 6 healthcare dollars spent [3]. Although the currently available pharmacological and interventional therapeutic options relieve symptoms and reduce adverse cardiac remodeling, they are for the most part incapable of addressing the fundamental pathology of irreversible heart tissue loss [4,5]. Innovative stem cell therapies that stimulate the regeneration of damaged cardiac tissue have the potential to profoundly modify the conventional treatment of CVDs [6-8].

There is growing evidence that cell types such as bone marrow mononuclear cells, mesenchymal stem cells (MSCs), and cardiac stem cells (CSCs) influence the injured heart via paracrine or regenerative pathways [9-11]. Human umbilical cord-derived mesenchymal stromal cells (HUC-MSCs), a common form of MSCs, have demonstrated great promise and have vast application potential in cell-based cardiac repair [12-14]. HUC-MSCs can be isolated from variable components of the umbilical cord, such as the Wharton’s jelly, cord lining, and the perivascular region [15-17].

Due to their capacity for self-renewal and multi-lineage differentiation into a variety of regenerative-promoting specialized cell types, HUC-MSCs have been intensively researched in this context [18,19]. Relatively recently, HUC-MSC banking utilizing approved processing, manufacturing, and storage techniques guarantees the global availability of high-quality, functioning, and well-characterized cells for application in clinical studies [20,21]. The stemness of HUC-MSCs might be affected by several passages and growth [14]. In addition to their immunomodulatory and anti-inflammatory properties, HUC-MSCs primarily exert their effects via paracrine mechanisms. They are suitable for use in allografts as their surface antigens are low, the risk of rejection of transplanted cells is low, and stringent matching requirements are not needed [22-24]. HUC-MSCs, in contrast to bone marrow-derived MSCs (BM-MSCs), lack the limitations of the invasive isolation methods and reduced proliferation and differentiation capability associated with donor age and comorbidities, which limit the therapeutic relevance of BM-MSCs. Furthermore, they are capable of self-renewal at a greater rate than BM-MSCs and lack the ethical concerns and tumorigenic/carcinogenic potential associated with embryonic stem cells [25,26]. Extensive animal research on HUC-MSCs has yielded encouraging results and has laid the groundwork for future clinical trials [19,27-29].

Clinical trials involving HUC-MSCs in the treatment of CVDs are controversial but encouraging. Some have yielded neutral results. Others were undertaken with small sample sizes. Moreover, several questions, such as the appropriate dose of stem cells that must be injected, the optimal mode of cell delivery, and the optimal delivery schedule to enhance recovery of heart function after myocardial infarction (MI) or heart failure, remain unanswered. Consequently, a meta-analysis was performed to determine the true impact of these cells. There is a lack of clinical evidence about the efficacy of HUC-MSC transplantation for patients with heart disease. This meta-analysis aims to synthesize evidence from published randomized controlled trials (RCTs) about the safety and efficacy of HUC-MSC transplantation for patients with heart failure and MI.

## Review

Methodology

The Preferred Reporting Items for Systematic Reviews and Meta-Analyses (PRISMA) statement standards were adhered to in the development of this review and meta-analysis.

Inclusion and Exclusion Criteria

Studies satisfying the following criteria were included in the present study: (1) parallel, randomized, controlled trials investigating the safety and efficacy of HUC-MSCs for cardiac repair such as heart failure and MI; (2) studies whose population included adult patients with symptomatic cardiac disease; (3) studies with HUC-MSC transplantation intervention; (4) studies with a control group taking placebo; and (5) studies reporting the mean change in left ventricular ejection fraction (LVEF), infarct size, six-minute walk distance, and functional status, adverse effects from baseline to the end of the follow-up period. We excluded studies that were not accessible in the English language, as well as thesis papers, conference abstracts, and studies for which the data were not retrievable for extraction and analysis.

Literature Search Strategy

A literature search of PubMed was conducted up to December 2022. For the sensitive search strategy, we used “clinical trial” as a filter for our search. We also used the MeSH database and the following search queries: “umbilical cord-derived mesenchymal stromal cells AND heart” OR “umbilical cord-derived mesenchymal stem cells AND ischemic heart disease” OR “Wharton’s jelly-derived mesenchymal stem cells AND acute myocardial infarction” OR “umbilical cord-derived mesenchymal stem cell AND coronary artery disease” OR “umbilical cord-derived mesenchymal stem cell AND heart failure.”

The titles and abstracts of the retrieved citations were checked by two authors. The process of eligibility screening was conducted in a two-step manner. The initial stage involved the screening of abstracts to determine their eligibility. Subsequently, the second stage entailed retrieving and screening full-text articles of the acceptable abstracts to ascertain their suitability for inclusion in the meta-analysis.

Data Extraction

The data extraction process was conducted by two authors independently via an online data extraction form. The data that were extracted encompassed the following components: (1) the design of the study; (2) the population that was studied; (3) the domains related to the risk of bias; and (4) the outcomes of the investigation, which comprised the mean change in the LVEF, the size of the infarct, the distance covered in a six-minute walk, the functional status, and any adverse effects seen. Disagreements were addressed through the authoritative input of the senior author.

Quality Assessment

Quality assessment of the retrieved RCTs was conducted according to the guidelines outlined in the Cochrane Handbook for Systematic Reviews of Interventions 5.1.0, which was last updated in March 2011. The quality assessment table supplied in Part 2, Chapter 8.5 of the same handbook was utilized for this purpose. The Cochrane risk of bias assessment tool encompasses several domains that are crucial for evaluating the quality of a study. These domains include sequence generation, allocation sequence concealment, blinding of participants and personnel, blinding of outcome assessment, incomplete outcome data, selective outcome reporting, and other possible sources of bias. The evaluation conducted by the authors is categorized as “Low risk,” “High risk,” or “Unclear risk” in terms of bias.

Subgroup Analysis

For the outcome of LVEF improvement, data are presented in two subgroups according to the follow-up period after HUC-MSCs transplantation: the first subgroup at 12-month follow-up and the second subgroup at six-month follow-up.

Assessment of Heterogeneity

The evaluation of heterogeneity encompassed a comprehensive examination of the forest plots, alongside the application of statistical measures such as I-square (I^2^) and chi-square tests. The chi-square test was utilized to assess the presence of statistically significant heterogeneity, while the I^2^ statistic was employed to quantify the extent of heterogeneity in the effect size. The assessment and interpretation of heterogeneity were conducted following the guidelines outlined in the Cochrane Handbook of Systematic Reviews and Meta-analysis (Chapter 9). In this manual, a critical value of less than 0.1 indicates considerable heterogeneity for the chi-square test. The I^2^ test is interpreted as follows: a range of 0-40% may not be of great importance, 30-60% may indicate moderate heterogeneity, and 50-90% may suggest substantial heterogeneity. The utilization of the random-effect model was implemented in cases where significant heterogeneity existed.

Data Synthesis

Improvement in ejection fraction (EF) was pooled as mean difference (MD) in a meta-analysis model in a random-effect model while readmission and mortality were pooled as risk ratio (RR) using the Mantel-Haenszel (M-H) method. The random-effect model was used due to the heterogeneity of some included studies while the fixed-effect model was used under the assumption that the included studies were homogeneous and comparable in terms of study design, quality, and measures of treatment effect. We used Review Manager 5.3 for Windows.

Publication Bias

According to Egger and colleagues [30,31], publication bias assessment is not reliable for less than 10 pooled studies. Consequently, we could not assess the presence of publication bias in our study by Egger’s test for funnel plot asymmetry.

Results

Search Results

**Figure 1:**
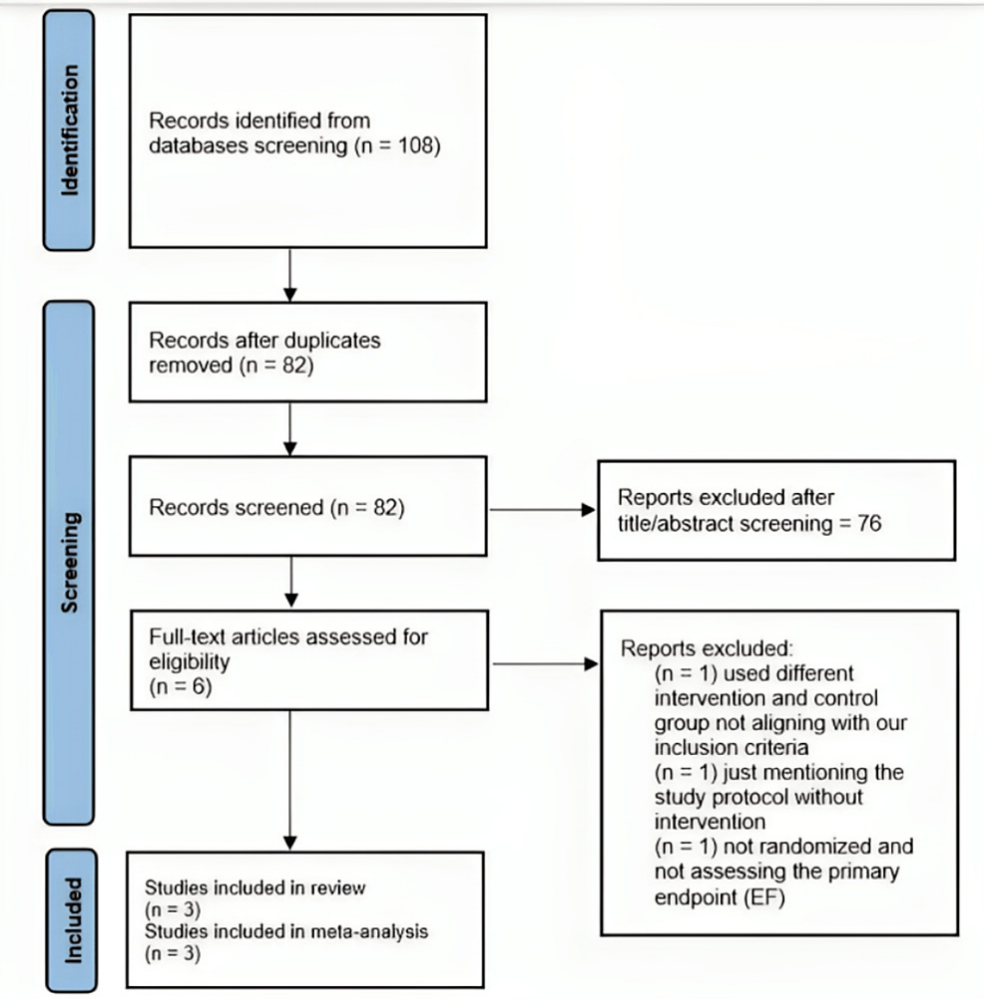
Preferred Reporting Items for Systematic Reviews and Meta-Analyses flow diagram.

A search conducted on PubMed utilizing the NCBI filter “clinical trial” yielded a total of nine clinical studies. After conducting the title and abstract screening process, six articles met the criteria for full-text screening. Among the available options, three clinical trials encompassing a combined cohort of 201 individuals were deemed suitable for inclusion in the final analysis. Three of the trials were excluded because the composition of the intervention and control groups did not meet the inclusion criteria outlined in our meta-analysis, as depicted in the PRISMA flow diagram (Figure 1). Table 1 presents a comprehensive overview of the included research, along with their primary findings. Meanwhile, Table 2 provides the baseline characteristics of the study populations involved in these investigations.

**Table 1:** Summary of the included studies. UC-MSCs: umbilical cord-derived mesenchymal stem cells; BM-SCs: bone marrow-derived stem cells; AMI: acute myocardial infarction; CHF: congestive heart failure

Summary of included studies								
Study	Design	Sample size			Population	Duration of follow-up post-transplantation (months)	Results	
		Intervention	Control	Total				
Gao et al. [32]	Double-blinded RCT	57	55	112	ST elevation acute MI patients	12	Intracoronary infusion of UC-MSCs is safe and effective in patients with ST elevation AMI which offers a clinically promising solution. Further investigation is needed to compare UC-MSCs to BM-SCs in the treatment of AMI	
								Zhao et al. [33]	Double-blinded RCT	30	29	59	Congestive heart failure patients	6	Compared with the control group, patients with intracoronary transplantation of UC-MSCs showed significant improvement in CHF symptoms and parameters. However owing to the short duration of observation and the limited number of patients, the efficacy and safety of the intervention need to be further explored	
																Bartolucci et al. [34]	Double-blinded RCT	15	15	30	Congestive heart failure patients	12	Compared with the control group, intravenous injection of UC-MSCs showed both safety and improvement of functional status and quality of life in CHF patients. Further testing through larger clinical trials is needed	
																								Double-blinded RCT	15	15	30	Congestive heart failure patients	6	Compared with the control group, intravenous injection of UC-MSCs showed both safety and improvement of functional status and quality of life in CHF patients. Further testing through larger clinical trials is needed	

**Table 2:** Baseline characteristics. The data have been represented as mean ± SD and p-value. I: intervention; C: control; N/A: not available; NS: non-significant; LVEF: left ventricular ejection fraction; ACEIs: angiotensin-converting enzyme inhibitors; ARBs: angiotensin receptor blockers

Baseline characteristics: Demographics									
	Gao et al. [32]			Zhao et al. [33]			Bartolucci et al. [34]		
	I	C	P-value	I	C	P-value	I	C	P-value
Age	57.3 ± 1.3	56.7 ± 1.7	0.79	52.90 ± 16.32	53.21 ± 11.46	0.106	57.33 ± 10.05	57.20 ± 11.64	NS
Men (N, %)	55 (94.8)	51 (87.9)	0.18	24	19	>0.05	12 (80.0)	14 (93.3)	NS
Smoking (N, %)	34 (58.6)	32 (55.2)	0.7	N/A	N/A	N/A	7 (46.7)	4 (26.7)	NS
Baseline LVEF (SD)	52.0 ± 0.9	51.1 ± 1.0	0.51	0.30 ± 0.045	0.28 ± 0.049	0.659	33.00 ± 6.18	31.49 ± 4.71	NS
Baseline characteristics: Comorbid diseases									
	Gao et al. [32]			Zhao et al. [33]			Bartolucci et al. [34]		
	I	C	P-value	I	C	P-value	I	C	P-value
DM (N, %)	17 (29.3)	14 (24.1)	0.52	N/A	N/A	N/A	5 (33.3)	7 (46.7)	NS
HTN (N, %)	33 (56.9)	26 (44.8)	0.19	N/A	N/A	N/A	7 (46.7)	8 (53.3)	NS
Ischemic CM (N, %)	N/A	N/A	N/A	13	4	>0.05	10 (66.7)	11 (73.3)	NS
Baseline characteristics: Medications									
	Gao et al. [32]			Zhao et al. [33]			Bartolucci et al. [34]		
	I	C	P-value	I	C	P-value	I	C	P-value
ACEIs/ARBs (N, %)	42 (72.4)	43 (74.1)	0.83	29	28	>0.05	15 (100)	15 (100)	NS
Aspirin (N, %)	58 (100)	58 (100)	1	N/A			14 (93.3)	9 (60.0)	0.031
Beta blockers (N, %)	42 (72.4)	48 (82.8)	0.18	29	28	>0.05	15 (100)	15 (100)	NS
Digitalis (N, %)	N/A			24	26	>0.05	4 (26.7)	1 (6.7)	NS

Quality of Included Studies

**Figure 2:**
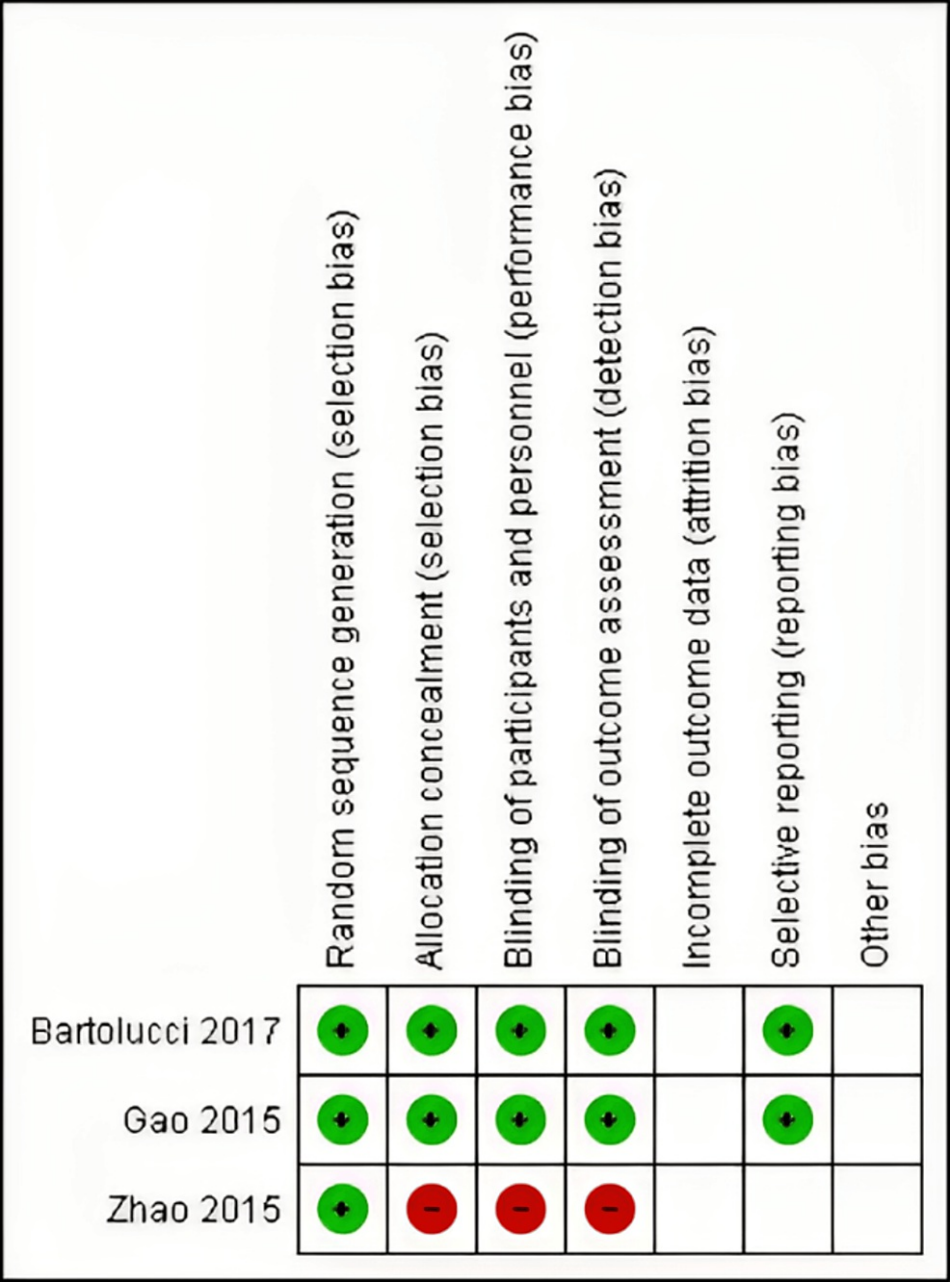
Risk of bias summary. Gao et al. [32], Zhao et al. [33], and Bartolucci et al. [34].

**Figure 3:**
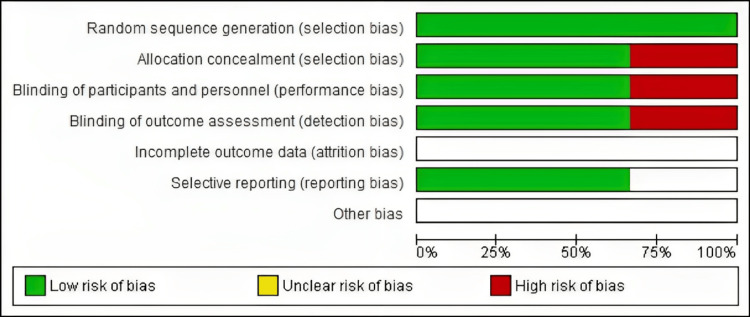
Risk of bias graph.

The studies included in the analysis exhibited a range of quality levels, as determined by the Cochrane risk of bias assessment method. The depiction of the quality evaluation domains of the research considered is presented in Figure 2 and Figure 3.

Efficacy Analysis (From Baseline to Endpoint)

**Figure 4:**
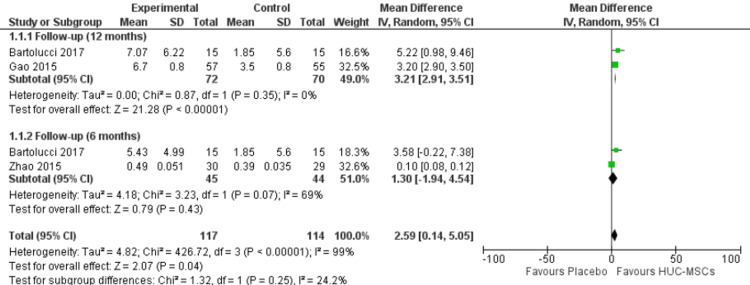
Forest plot of the change in left ventricular ejection fraction. Gao et al. [32], Zhao et al. [33], and Bartolucci et al. [34].

Left ventricular ejection fraction: Subgroup analysis of the two subgroups was performed according to the follow-up period after HUC-MSC transplantation (Figure 4). In the 12-month follow-up subgroup, the overall effect favored the HUC-MSC group over the placebo group (MD = 3.21, 95% confidence interval (CI) = 2.91-3.51, p < 0.00001); the pooled studies were homogenous (p = 0.35; I^2^ = 0%). In the six-month follow-up subgroup, the overall effect did not favor the HUC-MSC group over the placebo group (MD = 1.30, 95% CI = -1.94-4.54, p = 0.43); the pooled studies were heterogenous (p = 0.07; I^2^ = 69%).

**Figure 5:**
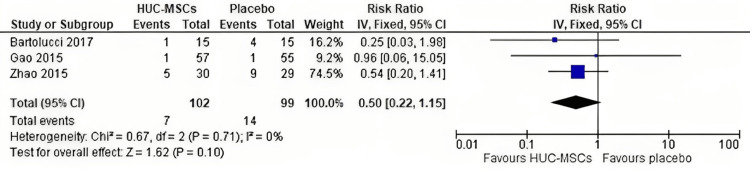
Forest plot of the risk of readmission. Gao et al. [32], Zhao et al. [33], and Bartolucci et al. [34].

Readmission: There was no statistically significant difference in readmission between the HUC-MSC group and the placebo group (RR = 0.5, 95% CI = 0.22-1.15, p = 0.10) (Figure 5); the pooled studies were homogeneous (p = 0.71; I^2^ = 0%).

**Figure 6:**
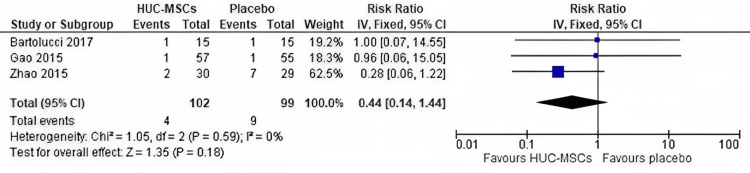
Forest plot of the risk of mortality. Gao et al. [32], Zhao et al. [33], and Bartolucci et al. [34].

Mortality: There was no statistically significant difference in mortality between the HUC-MSC group and placebo group (RR = 0.44, 95% CI = 0.14-1.44, p = 0.18) (Figure 6); the pooled studies were homogeneous (p = 0.59; I^2^ = 0 %).

Discussion

Summary of Main Results

Although numerous preclinical studies have demonstrated the advantages of HUC-MSC treatment for animal models with heart failure or MI [27,29], few clinical trials have explored the safety and efficacy of HUC-MSC therapy for heart failure and MI patients. Our meta-analysis demonstrated that transplantation of HUC-MSC into the heart may increase LVEF after 12 months. There is, however, no statistically significant difference between HUC-MSC and the control group in terms of readmission risk or mortality rates. As only two of the three studies in the meta-analysis enrolled patients with baseline heart failure with reduced EF, it is possible that the effect of HUC-MSCs on improving LVEF may be underestimated in our meta-analysis. It is also important to note that a statistically significant increase in LVEF does not necessarily correspond to a clinical benefit.

While the improvement in LVEF is a significant finding in the context of stem cell therapy for heart failure and MI, it is essential to bridge the gap between statistical significance and the clinical implications of this improvement. The clinical relevance of an increased LVEF should be assessed not only in terms of statistical values but also in its impact on patients’ lives.

Symptom alleviation: An increase in LVEF may correspond to a reduction in symptoms experienced by patients, such as breathlessness, fatigue, or exercise intolerance. It is crucial to investigate whether the improvement in LVEF translates into a noticeable reduction in these symptoms, which can significantly enhance a patient’s quality of life.

Quality of life: A paramount measure of clinical relevance is the impact on the overall quality of life. Assess whether patients who received HUC-MSC therapy report an improved quality of life, including their ability to perform daily activities, engage in social interactions, and experience an enhanced sense of well-being. Measuring quality of life may require standardized questionnaires or interviews.

Functional outcomes: In addition to LVEF, evaluating functional outcomes is vital. Patients’ capacity to perform physical tasks, such as the six-minute walk test, should be explored. An increase in LVEF might manifest as enhanced physical abilities, indicating a genuine clinical benefit.

Hospitalization rates: Examine whether there is any impact on hospitalization rates. A decrease in hospital readmissions could indicate that patients are experiencing fewer severe cardiac events, reflecting a tangible improvement in their health and a reduction in the economic burden of healthcare.

Long-term outcomes: Consider the long-term implications of improved LVEF. Determine whether this improvement translates into a better long-term prognosis, including reduced mortality and fewer adverse cardiac events. Assess the durability of the benefits and their continued impact on patients’ lives.

Duration of Follow-Up

The duration of follow-up following HUC-MSC transplantation varied between trials and appeared to have a significant impact on the therapeutic outcome. Gao et al. reported their findings at a 12-month follow-up while Zhao et al. reported at a six-month follow-up [32,33]. Bartolucci et al. reported their findings at both six-month and 12-month follow-up intervals [34]. Using subgroup analysis, a six-month follow-up period following transplantation of HUC-MSCs did not show a difference between the treatment and control groups. Nonetheless, a 12-month follow-up demonstrated an improvement in LVEF in the treatment group relative to the control group (MD = 3.21, 95% CI = 2.91-3.51, p < 0.00001) (Figure 4); the pooled studies were homogenous (p = 0.35; I^2^ = 0%).

Quality of Evidence

Because it is based on RCTs, the quality of this evidence is reliable. The techniques of search and the eligibility criteria were established. To prepare for this study, we used the PRISMA checklist and followed the Cochrane Handbook for Systematic Reviews of Interventions strictly.

Limitations

The limitations of this meta-analysis include limited data, with only three small-scale RCTs involving a total of 201 patients, potentially limiting the generalizability of findings. Heterogeneous follow-up durations across the included studies (six months and 12 months) resulted in varying results and made it challenging to draw definitive conclusions. A small number of studies prevented a reliable assessment of publication bias, potentially affecting the accuracy of the overall conclusions. The risk of bias in some included studies across different domains could introduce bias into the meta-analysis. Additionally, the analysis primarily focused on LVEF, readmission rates, and mortality rates, neglecting other crucial clinical outcomes such as quality of life and symptom improvement. Variations in patient characteristics across the included studies may impact treatment responses, and this heterogeneity was not extensively discussed. The longest follow-up period in the studies was 12 months, limiting the assessment of long-term effects and safety considerations, and the findings may not apply to a broader range of patients with heart failure and MI due to the specific characteristics of the study populations.

The economic considerations associated with stem cell therapy in the treatment of heart failure and acute MI are multifaceted. Stem cell interventions pose significant economic implications that warrant careful examination for feasibility and broader implementation. Key economic factors include the costs of cell acquisition and processing, where sophisticated laboratory techniques and quality control measures contribute substantially. Additionally, treatment administration costs, encompassing expertise, medical facilities, and specialized equipment, add to the economic considerations. Post-treatment, follow-up, and monitoring expenses play a crucial role in involving medical appointments, diagnostic tests, and potential imaging studies. Comparative cost-effectiveness analysis against standard treatments is vital for informed decision-making.

Future directions

As this meta-analysis contributes valuable insights into the efficacy of HUC-MSC therapy for heart failure and acute MI, it also points toward several promising avenues for future research. Future studies should extend the follow-up duration beyond the 12-month mark to assess the durability and sustainability of observed improvements and determine whether the initial increase in LVEF results in long-term clinical benefits, such as reduced mortality and fewer cardiac events. Investigating the impact of HUC-MSC therapy on specific patient subgroups, including different age groups, genders, comorbidity profiles, or disease severity, can guide more personalized treatment approaches. Incorporating robust quality-of-life assessments is essential to provide a comprehensive view of how HUC-MSC therapy affects patients’ daily lives, well-being, and overall satisfaction. Assessing the therapy’s impact on functional outcomes beyond LVEF, such as exercise tolerance, cardiac function, and the ability to perform daily activities, can shed light on the practical significance of LVEF improvements. Exploring the potential synergy of HUC-MSC therapy with other interventions, determining optimal dosage and timing, delving deeper into the mechanisms through which HUC-MSCs exert their effects, and monitoring patients for long-term safety and adverse effects are important research avenues. Focusing on patient-centered outcomes that matter most to patients and developing clinical guidelines and standardized protocols for HUC-MSC therapy will ensure consistency in treatment approaches and address ethical considerations as stem cell therapies evolve.

## Conclusions

Preclinical research utilizing HUC-MSCs to treat heart failure or MI has yielded provocative results, paving the path for their use in clinical trials. Our meta-analysis reveals that HUC-MSCs can enhance LVEF in patients with heart failure 12 months after transplantation. However, additional large-scale, randomized, double-blind studies are required to establish the safety and efficacy of HUC-MSC therapy in patients with heart failure and MI and to demonstrate whether statistical significance translates into clinical benefit.
